# More than support to court: Rape victims and specialist sexual violence services

**DOI:** 10.1177/0269758017742717

**Published:** 2017-12-06

**Authors:** Marianne Hester, Sarah-Jane Lilley

**Affiliations:** University of Bristol, UK; University of Bristol, UK

**Keywords:** Rape, sexual violence, victim needs, sexual violence services, ISVAs

## Abstract

This article explores the involvement of specialist sexual violence services, including Independent Sexual Violence Advisers (ISVAs), in supporting victims/survivors of rape and sexual abuse to engage with the criminal justice system (CJS) in England and Wales. The underpinning research, conducted in one area of England, included referral data from the police and key specialist sexual violence services, interviews with 15 victims/survivors of sexual violence in contact with the police and specialist services, and interviews with 14 practitioners from sexual violence and related services. We examine the complex needs of victims/survivors of sexual violence (who have experienced historical child sexual abuse, acquaintance rape or rape in the context of intimate partner abuse), how their needs differ and vary over time, and the ways in which these diverse and changing needs are met by specialist sexual violence services. Non-specialist agencies, such as statutory mental health services, are unable to provide similarly targeted responses. The research found that specialist sexual violence services play particularly crucial roles through the use of approaches that can be characterised as flexible, enabling, holding and mending. However, this important work could easily be lost in the current climate of local service commissioning, to the great detriment of victims/survivors of sexual violence.

## Introduction

Rape can have a devastating impact on every aspect of victims’/survivors’ lives and make them vulnerable to further episodes of sexual abuse or violence (McMillan and Thomas, 2009). There can be long-term physical, psychological and wider impacts of being the victim of rape and sexual assault that include post-traumatic stress disorder, depression, anxiety, inability to sleep and other effects, such as physical disability. There are also secondary effects, such as a reduction in victims’ ability to work or study, difficulties with forging new relationships or maintaining positive relationships with family and friends, or problems with their ability to care for others, their children, for example (McNaughton Nicholls et al., 2012: 21). In a study comparing mental health and general population samples in England and Wales, Khalifeh et al. (2014) found that individuals with severe mental health problems were 2.9 times more likely to have experienced sexual violence in the past year.

Before the late 1990s, specialist support for victims of sexual violence in England and Wales was mainly provided by Rape Crisis Centres (RCCs) situated in the voluntary sector (Westmarland and Alderson, 2013). Despite the name ‘Crisis’, RCCs have dealt with a large proportion of historical rape or childhood sexual abuse (CSA) cases. By 2015 there were 48 RCC services in England and Wales (Hawkins and Taylor, 2015).

Since 2000, attempts to improve victim treatment have also included provision of Sexual Assault Referral Centres (SARCs) and Independent Sexual Violence Advisers (ISVAs). There are now around 41 SARCs across the UK (Survivors Trust, 2015), largely funded by and based in the statutory health sector and which tend to support recent cases of rape and sexual assault and provide forensic examination. ISVAs are based on the existing Independent Domestic Violence Adviser (IDVA) model of specialist and independent victim-focused service provision (Robinson, 2009). First introduced in the mid 2000s, ISVAs have increased to at least 251 across England and Wales (Lea et al., 2015) and tend to be situated within SARCs or RCCs (Lea et al., 2015). Guidance on SARCs specifies that they should have ISVAs as part of their provision (Department of Health et al., 2009).

While government policy has acknowledged the importance of specialist sexual violence services such as SARCs, ISVAs and RCCS, the relationship between them is by no means clear, and different mixes of services with various funding streams have developed in different localities (Brown et al., 2010; Lea et al., 2015). Towards the end of our research, new commissioning processes were being considered for sexual violence services in the research locality with health commissioners questioning the nature and range of services, and their structures and governance. The commissioners had difficulty understanding the rationale for voluntary sector specialist sexual violence services, and were considering re-focusing sexual violence support within mental health services and the SARC. However, while mental health services provide support for victims of sexual violence, this does not necessarily involve specialist input (Westmarland and Alderson, 2013).

Health commissioners have ‘often overlooked rape crises, seeing them as “niche” or even irrelevant to health commissioning’ (Westmarland et al., 2013: 3266). Woody and Beldin (2012) point to the different philosophical perspectives of the sectors. Voluntary sector specialist sexual violence services are rooted in a feminist empowerment perspective that frames rape as a gendered, social and ‘whole-person’ issue, albeit with increasing professionalism and use of recognised psychological approaches. The mental health sector is rooted to a greater extent in the medical model and symptomatic approaches, which can also make it more difficult to focus on victim/survivor needs.

## Previous studies

An overview of the evidence on sexual violence support suggests that specialist sexual violence services and RCCs in particular score the highest in terms of helpfulness and victim satisfaction, in the UK and elsewhere (Brown et al., 2010). The few existing evaluations of SARCs have generally been positive, with victims/survivors liking the emphasis on examination by female staff, proactive follow up, advocacy and case tracking, and practical support (Lovett et al., 2004; Regan et al., 2008; Robinson, 2009). An evaluation of RCCs in the north of England (Westmarland et al., 2013), found RCC intervention led to notable improvements in victims’/survivors’ feelings of empowerment and increased control over their lives, and also a reduction in flashbacks and panic attacks.

The *Stern Review* (2010) into rape cases in England and Wales suggested that ISVAs are the most effective, cost-effective and affordable example of a reform to a system, making an enormous difference to how victims feel about what is happening to them as they process through the criminal justice system (CJS). Limited evidence from Crown Prosecution data also suggests that ISVAs may reduce the number of retractions by victims (Brown et al., 2010). Research from the US provides the clearest picture of the positive impact of specialist advocates such as ISVAs, showing that they lead to improved outcomes for victims, including reducing the number of negative responses from the police and health professionals, and buffering against the distress caused by the legal process (Campbell, 2006).

Research in England and Wales found ISVAs in both SARCs and RCCs deliver varied and important services going beyond criminal justice outcomes, both to individual victims (who saw ISVAs as essential for their own recovery) and to their multi-agency partners (e.g. by providing institutional advocacy) and can, therefore, add value to the local response provided to victims/survivors of sexual violence (Robinson, 2009). In an evaluation of the SARC in Cardiff (Robinson et al., 2009) the emotional support provided by ISVAs was explored further and three categories of such support were identified: crisis intervention, ongoing non-therapeutic support and advocacy, with the majority of victims receiving at least one of these types of support. In addition to providing emotional support to victims, the evaluation also found that in more than one-quarter of cases the ISVAs were known to have provided emotional support to someone other than the victim, usually a parent or sibling. This was a previously ‘hidden’ feature of the work of ISVAs (Robinson et al., 2009: 63).

The first UK-wide audit of ISVAs (Lea et al., 2015) provides details about the role of 146 ISVAs and their caseloads. ISVAs were again found to be a mainly female and well-qualified workforce, employed in a variety of roles including ISVA, children’s ISVA, specialist ISVA, IDSVA (dealing with both domestic and sexual violence) and Young People’s Advocate. In addition, they could be working as a Domestic Violence Strategic Coordinator, a Young People’s Violence Advocate Coordinator or an Independent Stalking Advocacy Caseworker. Services ‘always accessed’ by ISVA clients were found to include emotional support, help with raising their awareness of the CJS and process, assistance with raising awareness of their legal rights and support with court visits. ISVAs also signposted clients to a wide range of external services, including housing, counselling and sexual health services, but more work is needed to fully understand the role of the ISVA and the benefits thereof for victims/survivors of sexual violence (Lea et al., 2015).

The current study helps to fill some of the gaps in knowledge highlighted by Robinson (2009) and Lea et al. (2015) via a detailed look at the ISVA role(s), including emotional support, the link with other sexual violence services, and the ways in which the needs of victims/survivors are experienced by the victims/survivors themselves and how well they see these as being met.

## Methods

The research, located in one area of England, involved a multi-method approach to assess referrals, victim/survivor needs and agency responses. Ethical approval was granted by the University of Bristol and all interviewees gave written or verbal consent.

Referral data from the SARC and one of the RCCs (the largest providers of specialist sexual violence support in the location) were analysed for the period March 2013 to April 2014 to explore referral pathways between sexual violence and other services.

In-depth interviews were carried out with 15 victims/survivors (12 women and 3 men) and 3 of their mothers, to explore their experiences and the level and type of any support they had received from criminal justice agencies, specialist sexual violence services and other services. In two instances (one where the victim was only 15 years old), the mothers were present during the interview. The sample was obtained via the sexual violence services, who identified clients fitting the research criteria (i.e. experience of sexual violence, contact with the CJS and specialist sexual violence services) and contacted them by letter to ask if they would be willing to participate in the study. Where consent was given, the services provided the research team with contact details and a researcher contacted the (ex-) clients directly by telephone to explain the research and arrange an interview. Interviews lasted between one and two hours. All interviews were digitally recorded (with consent), and transcribed verbatim prior to analysis.

The involvement of the sexual violence services in referring the victims/survivors for interview is likely to have influenced the shape of the sample, although we do not know what a random sample of clients fulfilling the research criteria might have looked like. Of the 15 victims/survivors interviewed, nine had experienced CSA, three had experienced rape in a domestically abusive relationship and another three had experienced acquaintance rape/indecent assault. This is likely to be an oversampling of individuals with CSA experience. However, a large proportion of CSA victims would be expected and national statistics on RCCs indicate that 47% of service users were adult survivors of child sexual abuse (Rape Crisis, 2015). Historical cases may also be more successful in progressing to court (Hester and Lilley, 2016). All 15 victims/survivors in the sample had some contact with the CJS: 13 cases ended up in court, and 11 resulted in conviction. The victims/survivors in the 13 court cases were all supported by ISVAs. The sample, therefore, reflects ISVA and sexual violence service involvement, and is very different from a population rape sample where few would be expected to tell anyone about their experience, to have contact with the police or to proceed to court (Brown et al., 2010; Hester and Lilley, 2016).

Analysis involved reading and re-reading transcripts to identify themes and using framework grids to record summaries from the thematic analysis and linked quotes from participants (Ritchie and Lewis, 2003). The main themes identified were: type of case (historical CSA, domestic violence, acquaintance); disclosure; police involvement; Crown Prosecution Service (CPS) and court involvement; support from ISVA, IDVA and /or other services/agencies; impacts; and any other important features (e.g. compensation claim).

Semi-structured interviews were conducted with 14 practitioners to explore the work of the ISVAs within the different agency settings and to better understand the model of specialist sexual violence service provision across the research location. The sample included ISVAs, service managers and other key support workers from the five specialist sexual violence services in the locality and from Victim Support (non-specialist agency that provides advocacy support to victims/survivors of sexual violence). The interviews were digitally recorded with consent and transcribed verbatim. As before, practitioner interview transcripts were analysed for initial themes or concepts emerging from the data and to develop further categories.

## The research location

During the research period there were eight ISVAs employed in the research location, four full-time and four part-time, including a male ISVA and a specialist children’s and young person’s ISVA. A Life Enhancement Skills Adviser (LESA) worked as part of the ISVA team to specifically support victims with regard to practical issues. The ISVAs were based across different settings, including within the SARC and five voluntary sector projects (two RCCs, two domestic and sexual violence projects and a children’s and young persons’ project focusing on sexual exploitation). Funding had recently been secured for a three-year specialist sex worker ISVA post to be based within one of the RCCs. The specialist sexual violence services were funded by the local authority, NHS England, the Ministry of Justice, and charitable funders and the Police and Crime Commissioner (PCC) and overseen by a strategy group. The SARC and RCCs worked to Skills for Justice National Occupational Standards (Skills for Justice, n.d.).

## Findings

### Referrals

Referral data from specialist sexual violence services evidenced the complex interrelationship between these services in providing specialist support targeted at victim/survivor needs. As expected (Lea et al., 2015; Robinson, 2009), the SARC had the largest client group, mostly referred from the police (86%), and was also responsible for referring more clients to other services. In that sense, they were operating as a ‘hub’, as required by policy (Department of Health et al., 2009). One of the RCCs received the largest proportion of referrals from the SARC (35% of SARC clients), and also had considerable ‘internal’ referrals to its ISVA, LESA and specialist counselling services. There was also considerable cross-referral between sexual violence and other services, such as those offering IDVA support for domestic violence or ISVA support for children. However, while there were some well-established pathways between specialist services (e.g. between one RCC and the children’s and young persons’ project to accommodate 14–18 year olds requiring child-specific support) there was still a need to develop and refine some existing referral pathways, both into and between specialist support services, to make specialist care referrals less complicated and more efficient for statutory agencies such as the police. At the time of the research, work was underway to develop a unified monitoring system in order to more accurately record referral pathways and track all sexual violence cases reporting/seeking support in order to ensure victim/survivors did not ‘fall off the radar’ of the services that had supported them initially once they had been referred out to other services.

### Needs and perspectives of victims/survivors

The needs of victims/survivors varied a great deal at different stages of their often protracted ‘journey’ from victim to survivor as they moved through disclosure of the abuse, to reporting it to the police, and then on to the eventual hearing of the offender in court, coming finally to the post-court appearance recovery period. Needs were in some ways linked to the different ‘types’ of sexual violence experienced, such as CSA or rape in domestically abusive relationships (Hester and Lilley, 2016). The interview data suggested that specialist sexual violence services were able to ‘track’ the change in intensity and frequency of support required by a victim/survivor depending on where they were at in their journey. Usually, more intensive support was needed at the beginning, that is, immediately following referral, but once the victim/survivor was involved with a specialist service/ISVA, the level of support required, especially emotional support, tended to stabilise. Generally, less frequent contact was required during police investigation but, often, as the court trial drew near, the need for support increased again, to help the victim/survivor cope with the associated emotions and questions that arose directly prior to, and during the trial.

### Process of disclosing

For 11 of the victims/survivors interviewed, especially those who experienced CSA, the abuse happened many years earlier. Five of these 11 disclosed the abuse while they were still children – to their parents, other family members or to the police or social services – but without being believed or without abusers being prosecuted. At least five suffered mental breakdowns following disclosure or, alternatively, decided to disclose the abuse after experiencing a mental breakdown. For Joanna, who experienced CSA, ‘the world spiralled out of control’ when she told her mother, who then reported the abuse to the police. Fran only disclosed the abuse seven years after it had happened because she felt like she was ‘cracking up’ and became fearful of being out of the house. She initially disclosed the abuse to her partner, who in turn told her parents who then reported it to the police. As a result of reporting the abuse to the police both victims/survivors received specialist sexual violence counselling as well as support from both the LESA and ISVA, and reported that without the support of the ISVA (for them and their family members) they would not have felt strong enough to go to court.

Two interviewees had been referred by their GPs for generic health sector counselling/psychiatric support due to the mental health effects of the abuse, but found the mental health professionals did not focus on the sexual abuse. As a result, these interviewees preferred specialist sexual violence services where the sexual abuse was discussed directly. David, who experienced sexual abuse from his adoptive father, suffered a breakdown after coming across his abuser again some years after he had managed to leave. He disclosed the abuse to his GP and was referred for NHS psychological support, only to be told they ‘can’t deal with this’, leaving him without support at this time. When David later reported the abuse to the police, they referred him to the specialist sexual violence service, where he received specialist counselling over two years as well as support through the court process from the ISVA based in the service, whom he described as an ‘absolute rock’ who believed and understood his experiences. David stated that the emotional support he received probably saved his life:

if it wasn’t for (ISVA) I think…You know when you’re on a low point, and you start thinking to yourself, is it really worth you being here? And you feel like you’re banging against a brick wall. Honestly and you can quote me on this, I’ve told her this to her face: I really do not believe I’d a’ been here if it wasn’t for (the ISVA). (David)

Martine was referred by her GP to an NHS psychologist for depression before she ever disclosed the sexual abuse by her foster father. When she eventually disclosed the abuse to the psychologist, she was referred to a specialist sexual violence service where she obtained the specialist support she needed that enabled her to eventually report the abuse to the police. A flexible combination of psychological and specialist support helped her deal with the different needs she had over time. The support from the ISVA was particularly valuable initially in that it helped her to articulate and understand her experience:

(the ISVA) pointed out that it’s been an abusive relationship. Well I didn’t picture it as that…now I can talk to her and if I don’t understand something, I know she will find out or explain it in a way that I would understand so I realise that it’s not normal behaviour. (Martine)

Two interviewees had been reluctant to disclose the abuse as a direct result of the influence and/or threats of the abuser. Imelda was scared to report the domestic abuse and anal rape she suffered from her husband. When he smashed up the house she rang her parents, who reported it to the police. The police referred Imelda to the SARC who put her in touch with a specialist sexual violence service. It was the emotional support from an ISVA here that enabled her to eventually engage with the police:

they gave me like a few days to think about what I wanted to do and I met with that lady from [the sexual violence service] who talked through all the different options supporting me in making the decision to go and do like the video interview. (Imelda)

Alice, despite understanding the CJS and how hard it can be to obtain a conviction for rape within domestic violence relationships, reported the abuse to the police following support from the SARC ISVA to whom she had disclosed it:

(the ISVA) was brilliant. She talked me through it. I think we spoke for about an hour before she said, ‘Well I can’t let you go home now, knowing what I know, and knowing you got two kids…What do you want to do?’ And I said, ‘Well I’ve got to ring the police, haven’t I?’…she rang them for me and organised all that sort o’ thing. And that’s when it started. (Alice)

### The criminal justice process

All the victims/survivors interviewed had been in contact with the police, often more than once depending on the response they obtained. As mentioned earlier, the perpetrators in 13 of the 15 cases were prosecuted, with 11 cases resulting in conviction. All the victims/survivors in the court cases were supported by ISVAs, whom the interviewees deemed crucial to their progression through the CJS. They described a variety of ways in which the ISVAs had an empowering role, initially helping them (in a non-therapeutic way) to understand and articulate their feelings (in a safe, neutral space), then allaying their worries and fears about the criminal justice process, going on to dispel myths about the CJS and, in due course, reassuring victims/survivors of the positive role that can be played by agencies such as the police. An important part of this reassuring role involved explaining that decisions made by criminal justice agencies were based on evidential issues rather than whether or not the victims were believed. Another important element of the ISVAs’ role involved keeping the routes of communication open and active between the victim and/or their family and the police, in terms of the progression of the case, which for most had been a source of anxiety:

but [police officer] well he was murder really. He would never get back to your phone calls. [The ISVA] kept sending him emails, she was phoning him. And nothing…he should’ve let us know the days when he had to appear, for the bail, he should’ve let us know but he didn’t. And it was only [the ISVA] having to ring up different people to find out what was going on with the police. (Nancy)

Interviewees described an overall supportive relationship with the ISVA, one that was built on trust and honesty and one which was essentially consistent and flexible in order to meet whatever their needs were at the time (including signposting them to other services), and which worked to ‘hold’ them in the system:

[the ISVA] supported me through the system, always explained what would be happening. I used to get, can’t remember (whether) it was weekly or fortnightly phone calls from [the ISVA] to see how I was, just constantly keep checking in, if I was struggling with anything whether it was finances or, you know, feeling obviously you can’t cope anymore, it’s too much then she’d start putting me into other services, and getting that help for me. (Beth)

With regard to ISVA support for a court appearance:

she just stood by me and told me that everything’s gonna be fine and she was just a shoulder to cry on…I was absolutely crapping myself. I was being sick and everything…and she got me there. I just didn’t know what to expect when I went in…if it wasn’t for [the ISVA], like I say, I wouldn’t a’ been able to do it. (Fran)

I don’t think it would’ve ended up going to court without them. Or I’d have gone to court but I’d have probably ended up being an absolute wreck. I’d have been lost without them to be honest. (Kay)

without speaking to her I don’t think I’d have done it. (Imelda)

For our sample, the criminal justice process, from reporting to eventual court outcome, tended to take a long time, usually 18 months to 2 years, echoing Ministry of Justice figures on rape cases (Ministry of Justice et al., 2013). Some cases were drawn-out by the perpetrators themselves, who, for example: inevitably denied any sexual abuse so that cases were contested; claimed they had dementia so that psychological reports had to be made; or had juries overturned. Adjournments were described by one interviewee as ‘mental torture’. Thus, the consistency and ‘holding’ element of ISVA support throughout the whole process became a crucial factor in keeping the victim/survivor engaged in the criminal justice process.

Laura, who experienced CSA from a family member, found the police involvement quite negative due to a lack of communication during the two years the case was in process. The CPS eventually decided to take no further action, but the police did not tell Laura why. The length of the process ‘ripped her family apart’ and led to Laura being bullied at school. The negative experience of the police made the response of the specialist sexual violence service even more important. She received specialist child-focused sexual violence counselling that was ‘brilliant’, while the ISVA based in another specialist service explained and kept her informed about the criminal justice process. This latter ‘was a godsend’ given the lack of information otherwise.

For those victims/survivors who experienced rape as part of domestic abuse, a mixture of emotional and practical support from both ISVAs and IDVAs as well as other sexual or domestic violence support was crucial, with specialist sexual violence support being particularly important during the criminal justice process if the abuser was being tried for sexual offences, and then domestic violence support in the longer term.

### Process following the court case

At least six of the victims/survivors continued to receive support following the conclusion of their cases, largely to deal with the ongoing emotional fallout from the abuse they had suffered. Such support was provided mainly by the ISVA and specialist counselling services that victims/survivors were already in contact with. Martine was well supported through the court case by a female police officer as well as an ISVA. The result of the case was that the jury was unable to agree on the outcome. This had a detrimental effect on Martine’s mental wellbeing as she felt that she was not believed by the court and, as a result, ended up taking sick leave from work. At the time of interview, she was still receiving ad hoc emotional support from the ISVA:

when I got the verdict, it just turned my world upside down all over again. I felt really low, crying, I couldn’t sleep…[the ISVA] has been a great help really. I can talk to her when I’m stressed out. (Martine)

Fran ‘unravelled’ after her abuser’s court conviction and became violent towards her partner. However, the ISVA who had supported her through the court process did not have the specific skills to help her through this, and so referred her for support from one of the other specialist sexual violence services in the area, which was very positive:

all the support I’ve had was spot on. I didn’t even think I would get that much support as what I did. They were just all perfect. (Fran)

Four victims/survivors interviewed had been advised they could pursue criminal injuries compensation. However, none of the claims had been successful either because of time lapse or evidential problems (neither of which were the fault of the victim). This created renewed stress and mental health impacts for these victims/survivors. For Beth, whose case had failed to proceed to court, rejection of her compensation claim and how the decision was communicated felt like ‘another slap in the face’, resulting in her being re-referred for specialist counselling. After Georgia’s abuser was convicted, she was also advised she could claim compensation. However, incorrect information initially provided by the police to the compensation board resulted in her claim being rejected:

I’m still struggling to fight for the justice of the compensation. That in itself feels like an injustice. I set out initially with the injustice of the abuse happening in the first place and then and I’ve ended up with another injustice. They’re looking to deal with the anxiety (the compensation is) causing me for now, hopefully get that out the (way) so that I can deal with the counselling for the abuse…that’s getting in the way of getting the counselling for the problem in the first place. (Georgia)

## Specialist sexual violence services

Interviews with both practitioners and victims/survivors revealed a range of interventions delivered by specialist sexual violence services, with ISVAs playing a key part in the victim’s ‘journey’. Key aspects of the ISVA role included: advising, advocating, educating, informing, liaising, facilitating, supporting, exploring, listening and communicating. Many features of the ISVA role were similar across the different agencies/settings, with a ‘core’ service of both emotional and practical support at the different stages of the victim/survivor’s journey, whether or not they chose to take the criminal justice route. Reflecting the findings from the UK audit (Lea et al., 2015), the ISVAs worked in slightly different ways depending on the agency within which they were based, with some focused on a particular aspect of the criminal justice process. Generally, victim-focused and flexible support was offered before a victim/survivor reported the abuse to the police, once they had reported it, pre-court, during court and for a finite period post-court. ISVAs empowered, supported and advocated for victims, offering different types and levels of support and playing out key roles that we have characterised as ‘enabler’, ‘holder’ and ‘mender’. These roles are specific to specialist sexual violence services and based on need and empowerment.

### Flexibility and focus on victim/survivor need

The flexibility of the ISVA role, combined with the wider input of specialist support, enabled victims to access even the most informal types of support in a safe environment. ISVAs and other frontline support workers in specialist support services described how they provided a safe space in which victims could ‘offload’ in times of crisis, as and when needed, rather than having to wait for an allocated appointment. Something may trigger a stressful reaction or episode at any time, and having unrestricted access to an ISVA, whether face to face, at the end of the telephone or in a building nearby to ‘offload’ at crisis point, fulfilled a particular need for victims who only wanted a ‘safe’ outlet at points of crisis rather than ongoing counselling. This was where the location and 24-hour access to the SARC was an advantage.

The specific concerns of the individual victim/survivor shaped the type and level of emotional support provided by ISVAs and specialist services, including whether or not the abuse had been reported, how it was reported and who chose to report it. For instance, there might be emotional fallout where a family member had ‘taken charge’ of the situation to report the sexual abuse, and the ISVA had to re-shift control back to the victim/survivor:

and sometimes it hasn’t been their decision to ring the police, someone else has made that decision for them…and that’s when you get people who, ‘oh my god I don’t want to do this’…So, the immediate thing is to unpick and put the realities into that because it’s not like you see on TV. (Specialist service manager)

The ISVAs also offered a finite period of support to victims/survivors (and/or family members) post-court, again depending on the specific needs of the individual. Examples included: facilitating a prison visit post-trial to enable a victim to meet with the perpetrator (who was a family member); making a referral to Victim Support for Criminal Injuries Compensation applications (where appropriate or requested); and facilitating engagement or re-engagement with therapeutic or specialist counselling services.

Where necessary, ISVAs provided essential support to family members while ensuring that victims/survivors themselves remained the main focus of support and that their full emotional needs were being met:

so, you have the mum who is terrified about what is going to happen to her daughter in court, the daughter can pick up on that…so if the whole family is supported then the victim is supported. (Specialist service manager)

While support for the family proved invaluable to some of the victims/survivors we spoke to, practitioners emphasised the importance of not allowing the victim/survivor’s needs to be overtaken by the needs of the family members, thus compromising support to the victim.

### A ‘boundaried’ role pre-trial

A particular challenge voiced by practitioners was ensuring they provided victims/survivors with essential emotional support prior to and during a court case, but were not perceived to be ‘coaching’ a vulnerable victim or witness, which could be detrimental to the outcome of that case. In response to this increasingly recognised concern one specialist sexual violence service in the research location had recently developed specific guidance on issues regarding therapy prior to criminal proceedings. This Pre-Trial Therapy Protocol – used by all the agencies responsible either for the provision of therapeutic services, the investigation of crime (police) or the prosecution of offenders (CPS) – provided guidance for work with victims/survivors aged 14 years and over to ensure the police and the CPS were informed and could advise on issues that might mitigate against starting therapy. Therapy had to be client-led, on a one-to-one basis, with the same therapist, with accurate recording, and no direct questioning of the client about their experiences. The emotional support described by practitioners was consequently of a non-direct nature, with ISVAs focusing on what victims/survivors were thinking and feeling and how they may be behaving as a consequence:

the CPS have always understood how I work, so they’re not scared of what I am doing with the victims, I’m not corrupting the evidence, I can’t change their view of what happened, obviously that’s dangerous, what I can help them with is their fears, worries, concerns about what happens next, what their place is within the criminal justice system. (ISVA)

### The ‘enabler’

ISVAs played a key role in enabling victims to start and continue on their journey through the oft-protracted process of transition to survivor, whether or not this involved going through the CJS. The ISVAs invested time and effort in the early stages to help victims understand and articulate their feelings and concerns as only then could they start to identify their individual needs and signpost or facilitate access to relevant support services available within the community. The listening and communication skills of ISVAs were deemed essential in helping victims work through and understand their worries and fears as often victims did not articulate or did not know what they were concerned about. Through dispelling myths about the CJS or reassuring victims of the positive role that can be played by agencies such as the police, the ISVAs assisted and empowered victims to make clear and informed decisions about the best route to take.

### The ‘holder’

If a victim decided to report the abuse to the police and proceed to court, the ISVA’s key role was to ensure the victim remained within the CJS. Interviewees described this role as ‘keeping’ or ‘holding’ victims in a safe way in the run up to and throughout the court case. The role varied and could be very complex, depending on the needs of the individual. It included help with any ongoing issues that could potentially add external pressure or influence a victim’s decision to disengage from support services and/or withdraw from the criminal justice process. This might be, for instance, keeping routes of communication between the victim and/or their family and the police open and active, or providing a wide range of practical assistance with issues such as housing, education, benefits, employment and health.

The consistency of ISVA support was considered vital. Practitioners suggested this may be particularly important for younger victims. For example, someone who disclosed abuse at 12 years old might be closer to 14 years old by the time their case reached court and they are, therefore, likely to have gone through many changes during that time: in appearance, personality development and with regard to feelings about what happened. Generally, ISVAs worked with victims/survivors for anything from a few weeks to two years. The length of time spent supporting or ‘holding’ a victim/client was directly linked to capacity levels within criminal justice agencies and interviewees suggested that ‘holding’ was taking longer because police and CPS investigations were also taking longer. This was frustrating for victims/survivors, and also had resource implications for the specialist services.

### The ‘mender’

Practitioner interviews also highlighted the ‘mender’ role of ISVAs. Interviewees described the way in which the emotional fallout of sexual violence can ripple throughout the wider family. The dynamics of familial relationships may change or situations of conflict can often occur within the family. In cases like these, ISVAs would often do some sort of mediation work with the family in order to reconcile the needs and wishes of the victim/survivor and the wider family:

we did have a case of No Further Action and mam was really wanting to start it again…We did some mediation around that because they weren’t getting anywhere…the daughter wanted to get on with her exams, wanted to get on with her life, the mam just wanted to see him punished for what he’s done and they were just at loggerheads. (ISVA)

By facilitating communication between family members, ISVAs helped to mend damaged relationships that had become fractured or difficult, and thus further strengthened the support network around the victim/survivor.

### The role of ISVAs in achieving ‘justice’

The interview data suggested there is a scale or spectrum of perceptions of ‘justice’ or ‘success’ amongst victims/survivors in cases of sexual violence and abuse, depending on the individual victim/survivor, and that this can range from empowerment to disclose the abuse, through seeking help and/or justice, to obtaining a guilty verdict in court and, finally, to the issuing of long-term sentences for perpetrators. ‘Success’ for ISVAs can include: putting the right type of support in place; making victims/survivors feel believed and/or validated; reducing or eliminating fears that have disempowered victims/survivors or held them back; and empowering victims/survivors to seek criminal justice, make confident life choices and ‘recover’. Interviews with victim/survivors alluded to the fact that, for them, the criminal justice outcome is not necessarily the only type of ‘justice’ required or at least is not the most important part of the story. For some, once the focus of the court case ends, different problems can arise (e.g. fallout within the family). Instead, ‘success’ or ‘justice’ can often relate to a victim being able to move forward with their life and is, therefore, linked more to their emotional wellbeing and ‘recovery’ than to a court outcome. For some, it is not the guilty verdict or custodial sentence that is important; achieving ‘justice’ might involve some sort of external validation of what happened to them, that is, hearing the perpetrator openly admit their guilt or having the perpetrator named and shamed in public. Thus, ISVA support can lead to ‘success’ in terms of helping victims/survivors acquire the emotional support they need to recover, delivering an alternative or broader type of justice that goes beyond the formal CJS. It could be argued, then, that the most important impact for victims/survivors and, thus, the success of ISVAs, might be measured as part of the whole justice *process* rather than the criminal justice outcome, that is, success in terms of increased criminal convictions for perpetrators of sexual violence and abuse.

## Discussion

As shown, specialist sexual violence services were crucial to the victims/survivors in providing a mix of specialist counselling (as an adult or young person), emotional support for court appearances, practical help and linkage of individuals with other agencies. Crucially, the specialist services were able to provide a changing mixture of targeted support as and when the victim’s/survivor’s needs changed, for instance increasing counselling support when they were feeling more depressed or suicidal, and providing ISVAs to support them through the often drawn-out criminal justice process.

Echoing the work of Campbell et al. (2001), where victims/survivors were not met with belief and understanding (e.g. from police or health professionals), or their needs were neither addressed nor met (e.g. court outcomes and process, compensation) this could have a devastating impact, with individuals tending to feel re-victimised and re-traumatised. The specialist services were able to support victims/survivors through such crises, providing empowering support and different forms of counselling input at different points of the victim/survivor journey.

Westmarland et al. (2012) point out that while it is impossible to remove the experience of sexual violence from an individual’s past, the individual will feel less distress and be less prone to post-traumatic stress the greater the perceived control they have over their present circumstances and over the recovery process. The emphasis on empowering victims/survivors to make their own decisions and progress at their own pace, identified by both our victim/survivor and practitioner interviewees, provided this crucial aspect of support.

Previous studies suggest the health impacts of sexual violence are greater for victims/survivors who have not disclosed the abuse or had it acknowledged (Clements and Ogle, 2009). At the same time, for victims of sexual violence, unlike other victims of trauma, the reception and response they get is particularly crucial (Ahrens et al., 2010). Receiving a negative response can be very detrimental. Moreover, disclosing the abuse may bring back memories and feelings associated with it to such an extent that mental breakdown may result. Disclosure is, therefore, risky, as the potential costs of telling someone include: loss of privacy; disapproval; economic pressures; not being believed; stress and anxiety; and risks to personal safety (Brown et al., 2010). The 15 victims/survivors in our sample experienced disclosure of the abuse in both negative and positive ways that profoundly shaped their ‘journey’ from victim to survivor and it was found that when the police responses were inconsistent and at times negative, the specialist sexual violence services provided the only ‘safe space’ (Brown et al., 2010) where disclosure and support tended to be consistently positive.

In contrast to the statutory mental health sector, where intervention is usually time limited with waiting lists and there is no targeted support (NatCen, 2015), the strength of the specialist sexual violence services and ISVAs was their flexibility and ability to target specific needs as and when required, using the skills of ‘enabler’, ‘holder’ and ‘mender’. This was underpinned by the ISVAs’ detailed knowledge and understanding of the specific impacts of sexual violence and how sexual violence impacts individuals and families, which was combined with a range of skills and roles within and across services and the possibility of quick referral between them. In this sense, the specialist services, with the mix of RCCs, SARC and ISVAs, played a unique role in enabling victims/survivors of sexual violence and abuse to seek help and/or justice by targeting their individual needs.

## Conclusion

As we alluded to in the introduction, in the new world of commissioning of sexual violence support services, the flexibility and variety of input provided by specialist sexual violence services may appear confusing to commissioners who are working within more limited statutory cultures and guidance. While the NHS guidance for commissioners, *Public Health Functions to be Exercised by the NHS Commissioning Board. Service Specification No. 30: Sexual Assault Services* (NHS Commissioning Board, 2012), acknowledges the importance of specialist sexual violence provision and the positive effect in terms of cost savings, only SARCs are mentioned as ‘models’ of sexual violence services. In contrast, the model of response for victims/survivors of sexual violence in the research location involved a range of different services (including the SARC, RCCs, ISVAs and DV services) for victims/survivors who had differing and complex needs. This was found to be a strength rather than a weakness (and see Brown et al., 2010; Westmarland and Alderson, 2013). Indeed, our work suggests that commissioning of effective sexual violence services requires specific consideration of the complex and changing needs of victims/survivors; such needs are most likely to be met by a closely linked network of a range of specialist providers. While the NHS guidance in *Public Health Functions to be Exercised by the NHS Commissioning Board. Service Specification No. 30: Sexual Assault Services* emphasises the key role of the SARC as the main gateway or ‘hub’ from which victims/ survivors can access appropriate care pathways, the voluntary sector specialist sexual violence services are positioned only as ‘sexual violence counselling providers’ (NHS Commissioning Board, 2012: Appendix 1) within care pathways. However, this does not reflect the much wider range of support provided by these voluntary sector services in the research location (or elsewhere, see Robinson, 2009). Commissioners thus need to be made aware of the actual roles and input provided by voluntary sector sexual violence services. Otherwise, statutory services (SARCs and NHS mental health services) will have a much more limited focus, with much poorer support for victims/survivors as a result.
